# Need for normalization: the non-standard reference standard for microvascular invasion diagnosis in hepatocellular carcinoma

**DOI:** 10.1186/s12957-018-1347-0

**Published:** 2018-03-07

**Authors:** Hang-Tong Hu, Zhu Wang, Ming Kuang, Wei Wang

**Affiliations:** 1 Department of Medical Ultrasonics, Institute of Diagnostic and Interventional Ultrasound, The First Affiliated Hospital of Sun Yat-Sen University, NO.58 Zhongshan Road 2, Guangzhou, 510080 People’s Republic of China; 2 Department of Hepatobiliary Surgery, The First Affiliated Hospital of Sun Yat-Sen University, Guangzhou, China

**Keywords:** Hepatocellular carcinoma, Microvascular invasion, Pathological examination

## Abstract

**Background:**

Preoperative microvascular invasion (MVI) assessment in hepatocellular carcinoma (HCC) is one of the current research focuses, with studies reporting controversial results regarding MVI-associated risk factors. As a possible source of bias, reported MVI rate (percentage of MVI-positive patients) varies a lot among studies. Pathological examination should have been the golden criteria of MVI diagnosis, but no standard and generally adopted pathological examination protocol exists.

**Methods and results:**

It is highly possible that underestimated pathological diagnosis of MVI exists. We present two likely examples to stress the problem and indicate the root of the problem partially being an unreliable pathological examination. Results of studies basing on unreliable reference standard can be less convincing and even misleading, which is the most basic and fundamental problem in this research field.

**Conclusion:**

There is an urgent need to settle the disputes regarding pathological sampling, microscopy, and reporting, in order to promote future academic exchange and consensus development on MVI assessment. Several concerns about pathological MVI assessment should be focused on in the future research as we put up in the review.

## Background

MVI refers to tumor invasion of vessels lined by endothelial cells, which substantially worsens the prognoses of HCC patients [1]. Successful preoperative assessment of MVI may change how the patient is managed and improve survival [2]. Factors reported to be associated with MVI risk in the previous studies are mixed and even controversial, tumor size for example [3–6]. As a possible source of bias, MVI rate varied significantly among studies, which is from 7.8 to 74.4% [3, 4]. The most frequently reported MVI-associated risk factors are alpha-fetoprotein (AFP), tumor size, tumor number, and histological differentiation [1, 4, 7, 8]. And preoperative anti-tumoral treatment such as trans-arterial-chemoembolization (TACE) or systemic chemotherapy may also have impact on MVI presence, which has not yet been thoroughly studied. All these factors considered, what would have caused the varied MVI rate?

Pathological examination is the reference standard for MVI diagnosis. However, in our experience, this gold standard is not always reliable in the clinical practice. The overall MVI rate of the retrospective and prospective database in our center is approximately 26 and 50%, respectively. And the previous negative results can also be altered by re-sampling of preserved surgical specimens, or intended for re-microscopy by a more experienced pathologist.

## Sampling

**Fig. 1:**
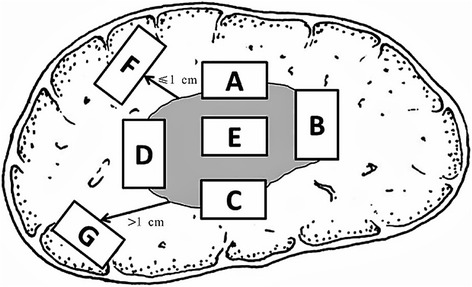
The “7-point baseline sample collection protocol” in China. (1) At least four specimens were located at 12 (A), 3 (B), 6 (C), and 9 (D) o’clock at the junction of the tumor and adjacent liver tissues; (2) at least one specimen at the intratumoral zone (E); (3) adjacent peritumoral liver tissues (F, ≤ 1 cm from the tumor capsule) and distant peritumoral liver tissues (G, > 1 cm from the tumor capsule) or the tumor margin [9]

A seldom previous study on preoperative MVI assessment has reported the pathological sampling protocol in detail. As a matter of fact, no referenced protocol exists until 2015 as the “7-point baseline sample collection protocol”, established by a cohort of Chinese pathologists (Fig. 1) [9, 10]. The protocol has put forward basic sampling requirements for MVI diagnosis, the detection reliability of which has not yet been verified. No further recommendation has been made regarding tumor size, shape, and location of HCC nodes, or the so called suspicious lesions suggested as sampling focus. And the protocol has not yet been generally adopted, at least in the other regions beyond China.

Kim et al. [11] revealed that MVI rate differed significantly between anatomical and un-anatomical surgical resection specimen. MVI was also reported to be detected more often in the site of tumor “protruding” [12] or capsule absence [8]. Thus, intended sampling considering these aspects should make a difference in pathological MVI detection rate.

## Microscopy

Most previous studies were observational and retrospective, in which MVI status could have been less routinely reported, let alone with specific staining methods and intended microscope inspection. Special immunohistochemistry is needed to differentiate vascular nature of which MVI invades, for example CD34 (vascular endothelium), smooth muscle α-actin (vascular smooth muscle), elastic fibers of vessel wall and D2–40 (lymphatic endothelium) [10, 13]. Iguchi et al. [14] revealed that MVI cell count of more than 50 and multiple-invaded vessels were indicators of poor prognosis. Relationship of tumor cells nests and the vascular wall was also reported to be associated with tumor recurrence and overall survival in patients with HCC after R0 liver resection [15]. Thus, invaded vessel type, tumor cell count, invaded vessel count, and relationship of tumor cells nests and the vascular wall should be recorded intentionally, which was less often the routine procedure in clinical practice.

## Discussion

Insufficient sampling and underreporting from the pathologists can be a general problem in the previous studies, which may have led to the possibly underestimated MVI rate. No direct evidence can be provided on this issue, while here we offer two likely examples for a brief glimpse into the problem. One is Huang et al. [16] and the colleagues reporting significantly different MVI rate (25.3 vs. 32.5%, *p* < 0.001) in two similar cohorts (Table 1). Applying the same eligibility criteria in the same ethnic group, the discovery and validation cohorts in the study are similar in a variety of aspects regarding age, gender percentage, HCC etiology, liver cirrhosis, Barcelona Clinic Liver Cancer (BCLC) stage, mean tumor size, tumor number, tumor encapsulation type (complete or none), histological grade, and AFP level, except for types of resection (anatomic or non-anatomic) (*p* = 0.046). Surgical approach was indicated to have no influence on MVI rate in the study by Shindoh et al [17]. With the most frequently reported MVI-associated risk factors considered, the difference in MVI rate seems explainable only with the possible different sampling methods.

**Table 1:** Comparisons between the two cohorts reported by Huang et al.

Features	Discovery cohort	Validation cohort	*p* value
Age (year, median (range))	53.0(10–86)※	53.0(12–92)※	1.00
Male (*n/N*)	1305/1540	530/630	0.78
HBV (*n/N*)	1255/1540	527/630	0.25
Liver cirrhosis (*n/N*)	1268/1540	512/630	0.62
BCLC stage (*B/N*)	154/1540	59/630	0.73
AFP > 200 ng/dL (*n/N*)	593/1540	259/630	0.28
Mean tumor size (cm, mean ± SD)	5.3 ± 3.5	5.5 ± 3.7	0.23
Patients with multiple tumors (*n/N*)	199/1540	78/630	0.81
Encapsulation (complete/*N*)	733/1540	294/630	0.74
Tumor differentiation (III–IV/*N*)	421/1540	171/630	0.97
Types of resection (anatomic/non-anatomic)	1222/318	475/155	< 0.05
MVI (*n/N*)	389/1540	205/630	< 0.05

Statistical analysis was performed using the MedCalc version 14.12.0 software program (MedCalc Software bvba, Ostend, Belgium)

*BCLC* Barcelona Clinic Liver Cancer stage, *AFP* alpha-fetoprotein, *MVI* microvascular invasion

※Data of mean and range were transformed into mean ± SD approximately with SD = 1/4(upper range–lower range)

Another example is the MVI rate (26.6 vs. 74.4%, *p* < 0.001) of two cohorts (Table 2), one reported by Qiao et al. [18] as the external validation for an HCC prognostic system, and another by Cucchetti et al. [4]. The two cohorts are derived from the same ethnic group from the University of Bologna, with possible overlapping population. They are different regarding percentage of patients with liver cirrhosis (10.0 vs. 79.2%, *p* < 0.001), CTP score (percentage of class C, 9.2 vs. 0%, *p* < 0.001), and AFP level (percentage of patients with AFP greater than 400 ng/ml, 23.5 vs. 10.0%, *p* < 0.001). Liver cirrhosis and CTP score have been reported to have no significant association with MVI risk by all previous studies. With a higher AFP level, the cohort in Qiao et al. presents an even lower MVI rate than that in Cucchetti et al. did. We notice that Cucchetti et al. included 20.0% of patients who have undergone liver transplantation, compared with no transplantation in Qiao et al. Thus, the possible different sampling methods resulted from the two different surgical procedures can be the most likely cause of different MVI detection rates.

**Table 2:** Comparisons between the two cohorts reported by Cucchetti et al. and Qiao et al.

Items	Cohort in Cucchetti et al.’s	External validation cohort in Qiao et al.’s	*p* value
Origin of patients	University of Bologna (1999 to 2008)	University of Bologna (2000 to 2011)	–
Age (years, mean ± SD)	62.8 ± 9.9	63.5 ± 9.4	0.40
Male (*n/N*)	193/250	227/293	0.98
HBV (*n/N*)	65/250	68/293	0.51
HCV (*n/N*)	164/250	202/293	0.47
Mean tumor size (cm, mean ± SD)	3.7 ± 1.8	3.9 ± 2.1	0.24
Moderate or poor tumor differentiation (*n/N*)	174/250	198/293	0.68
Patients with multiple tumors (*n/N*)	55/250	60/293	0.75
Liver cirrhosis (*n/N*)	250/250	232/293	< 0.05
CTP score (class C) (%)	9.2	0	< 0.05
AFP > 400 ng/ml (*n/N*)	25/250	69/293	< 0.05
MVI (*n/N*)	186/250	78/293	< 0.05

Statistical analysis was performed using the MedCalc version 14.12.0 software program (MedCalc Software bvba, Ostend, Belgium)

*CTP score* Child Pugh score, *AFP* alpha-fetoprotein, *MVI* microvascular invasion

## Conclusion

It is highly possible that underestimated MVI rate does exist; if not, there should be factors impacting on MVI risk to an extent greater than any of the crucial factors already known. Otherwise, selection bias would have been a major limitation of related studies. We think the most convincing way to put this issue into a verdict, as a suggestion for future research, is to conduct studies on how much intended pathological sampling and microscopy can improve MVI detection rate, in an effort to develop a standardized pathological examination and reporting protocol.

The “7-point baseline sample collection protocol” provides us with a basic framework, which needs improvement and further refinement. In order to achieve the highest MVI detection rate with a clinically feasible pathological examination protocol, future studies need to work on the following issues:

1. Considering the diverse size, shape and location of HCC nodes, (1) Is MVI clinically important when tumor size or number exceeds a particular threshold (for example, 5 cm in diameter or 3 nodes)? (2) What is the optimal sampling distance from the tumor boundary: 1 cm or 2 cm? (3) Which section to choose for sampling: transversal or longitudinal? (4) Is multiple-section sampling necessary? If it is, what is the optimal number of sampling sections? and (5) Is particular site sampling decisive, for example, site of tumor “protruding” or capsule absence?
2. As for microscopy and reporting, (1) What is the optimal number of slices per block for inspection? (2) What vessels invaded by MVI are significantly associated with prognosis: hepatic vein branches, portal vein branches, hepatic artery branches, or bile ducts? (3) How to record invaded vessel count: sum or average count in a slice serious, or the one with the most vessels invaded? (4) How to record tumor cell account: count in one vessel section, sum count in the vessel of a slice serious, or average count by vessel sectional area? and (5) further inspection into the association of relationship between MVI cell nests and the vascular wall with tumor prognosis.

With the disputes of pathological MVI diagnosis settled, academic exchange between studies on preoperative MVI assessment would be practicable and clinically valuable, and related guideline and consensus development would be greatly promoted, in hope of better management of HCC patients and improving survival.
